# Genomic prediction based on selective linkage disequilibrium pruning of low-coverage whole-genome sequence variants in a pure Duroc population

**DOI:** 10.1186/s12711-023-00843-w

**Published:** 2023-10-18

**Authors:** Di Zhu, Yiqiang Zhao, Ran Zhang, Hanyu Wu, Gengyuan Cai, Zhenfang Wu, Yuzhe Wang, Xiaoxiang Hu

**Affiliations:** 1 State Key Laboratory of Animal Biotech Breeding, College of Biological Sciences, China Agricultural University, Beijing, China; 2 National Research Facility for Phenotypic and Genotypic Analysis of Model Animals (Beijing), China Agricultural University, Beijing, China; 3 National Engineering Research Center for Breeding Swine Industry, South China Agricultural University, Guangdong, China

## Abstract

**Background:**

Although the accumulation of whole-genome sequencing (WGS) data has accelerated the identification of mutations underlying complex traits, its impact on the accuracy of genomic predictions is limited. Reliable genotyping data and pre-selected beneficial loci can be used to improve prediction accuracy. Previously, we reported a low-coverage sequencing genotyping method that yielded 11.3 million highly accurate single-nucleotide polymorphisms (SNPs) in pigs. Here, we introduce a method termed selective linkage disequilibrium pruning (SLDP), which refines the set of SNPs that show a large gain during prediction of complex traits using whole-genome SNP data.

**Results:**

We used the SLDP method to identify and select markers among millions of SNPs based on genome-wide association study (GWAS) prior information. We evaluated the performance of SLDP with respect to three real traits and six simulated traits with varying genetic architectures using two representative models (genomic best linear unbiased prediction and BayesR) on samples from 3579 Duroc boars. SLDP was determined by testing 180 combinations of two core parameters (GWAS P-value thresholds and linkage disequilibrium r^2^). The parameters for each trait were optimized in the training population by five fold cross-validation and then tested in the validation population. Similar to previous GWAS prior-based methods, the performance of SLDP was mainly affected by the genetic architecture of the traits analyzed. Specifically, SLDP performed better for traits controlled by major quantitative trait loci (QTL) or a small number of quantitative trait nucleotides (QTN). Compared with two commercial SNP chips, genotyping-by-sequencing data, and an unselected whole-genome SNP panel, the SLDP strategy led to significant improvements in prediction accuracy, which ranged from 0.84 to 3.22% for real traits controlled by major or moderate QTL and from 1.23 to 11.47% for simulated traits controlled by a small number of QTN.

**Conclusions:**

The SLDP marker selection method can be incorporated into mainstream prediction models to yield accuracy improvements for traits with a relatively simple genetic architecture, however, it has no significant advantage for traits not controlled by major QTL. The main factors that affect its performance are the genetic architecture of traits and the reliability of GWAS prior information. Our findings can facilitate the application of WGS-based genomic selection.

**Supplementary Information:**

The online version contains supplementary material available at 10.1186/s12711-023-00843-w.

## Background

The genomic prediction (GP) method proposed by Meuwissen et al. [1] is commonly used to predict breeding values based on high-density genome-wide single nucleotide polymorphisms (SNPs) and has been widely used in farm animals [2–4], plants [5–7], and human disease risk models [8]. Compared with pedigree-based breeding methods, in pigs the advantage of genomic selection is primarily reflected in the improved accuracy of genomic estimated breeding values (GEBV) rather than shorter generation intervals as in dairy cattle, since pigs and dairy cattle have different breeding characteristics. Therefore, improving prediction accuracy is the main objective of pig breeding. Prediction models and genetic markers are the two core elements for improving the accuracy of GP.

Genomic best linear unbiased prediction (GBLUP) is a commonly used prediction model that is based on a linear mixed model, which assumes that the effects of all the genetic markers contribute to phenotypic variation, and follow the same normal distribution [9, 10]. In contrast, Bayesian Alphabet models may be more adapted to the real situation, as most of them consider only a proportion of markers that contribute the genetic variance and as the markers have independent variances that follow a specific distribution [11, 12]. In addition, incorporating prior information into the basic model is more effective by making the model more biologically specific. Some studies have reported that the prediction accuracy of the GBLUP model based on pre-selected markers could be improved by increasing the weight of significant genome-wide association study (GWAS) markers in the genomic relationship matrix (GRM) [13, 14] or by combining chip data and panels of significant markers [15, 16]; moreover, fitting significant markers as separate variance components in the GBLUP model (i.e., multiple genomic relationship matrices-GBLUP) could also increase prediction accuracy [17, 18].

From the perspective of genetic markers, the degree of linkage disequilibrium (LD) between markers and the causal variant has a major impact on prediction accuracy. With the decline in sequencing costs and the emergence of low-coverage sequencing (LCS) strategies, it is now possible to use whole-genome sequencing (WGS) data for GP. The higher marker density in WGS data could increase the LD between markers and quantitative trait loci (QTL), in addition to enhancing the opportunity to directly capture the causal variant, which may consequently increase the overall accuracy of GP, as reported by several simulation studies [19–21]. However, the results obtained on real data remain ambiguous. For example, one study reported only a slight improvement in prediction accuracy when using imputed WGS data compared to using the 80K SNP chip in combined pig populations [22]. Another study showed that the use of WGS data did not increase the accuracy of GP for milk production and reproductive traits in Holstein–Friesian cattle [23] and for commercial traits in pigs when the sample size was limited [24]. There are two possible reasons for these findings. First, most WGS-based GP models are based on markers that are imputed from low- or high-density SNP arrays [25, 26]. The accuracy of this method is influenced by the quality of the reference panel and the genetic relationship with the target population [27], which can interfere with the genotyping quality and further critically impact the prediction accuracy. A second explanation is that massive noisy markers that are not in LD with any causal variant, were incorporated in the WGS data since the increase in SNP density is at random. Under this assumption, a large proportion of the variants in low LD with causal variants should be excluded when using millions of variants from WGS data.

Previously, we reported LCS genotyping analyses that can yield millions of highly accurate SNPs in pigs [28]. In the current study, we focused on marker selection and our aim was to identify the benefits of using WGS data for GP in a pure Duroc boar population. Toward this end, we propose a marker selection method called selective linkage disequilibrium pruning (SLDP) for selecting a subset of variants from LCS data based on GWAS prior information to improve prediction accuracy. The GBLUP and BayesR models were used in GP analyses for three real traits in pigs and six simulated traits with different genetic architectures. As a control, we simulated a series of SNP panels that were obtained using other genotyping techniques, including two commercial SNP arrays and genotyping-by-sequencing (GBS) [29], which were used in our earlier work [30].

## Methods

### Populations and phenotypes

In total, 3579 Duroc boars born between 2011 and 2016 were sampled from one nucleus farm provided by the Guangdong Wen’s Foodstuff Group (Guangdong, China), which included some boars from previous studies [28, 30, 31]. Phenotypic data included age at 100 kg live weight (AGE), backfat thickness (BF), and total teat number (TTN), representing quantitative traits with three distinct genetic architectures. In this population, AGE is a typical “infinitesimal trait” (i.e., no major QTL could be detected after long-term artificial selection), whereas TTN is mainly affected by several major QTL on *Sus scrofa* 11.1 chromosome (SSC)7 and BF is a transitional quantitative trait affected by several major loci and many minor gene interactions [28, 32, 33]. The trait statistics are summarized in Table 1 and Additional file 1: Fig. S1.

**Table 1:** Summary of the populations, number of animals used in the analyses, and means/medians of the phenotypic traits

Phenotype	Population	Individuals	Mean ± standard deviation	Median
AGE	Discovery	1000	154.66 ± 9.01	154.48
	Training	2108	155.07 ± 9.43	155.01
	Validation	441	153.81 ± 9.41	154.06
BF	Discovery	1000	10.94 ± 2.36	10.61
	Training	2107	10.98 ± 2.17	10.77
	Validation	441	10.76 ± 1.92	10.64
TTN	Discovery	1000	10.70 ± 1.05	11.00
	Training	2108	10.71 ± 1.07	11.00
	Validation	441	10.76 ± 1.03	11.00

The discovery population was used in the GWAS to select significant SNPs. The training population was used to optimize the parameters of SLDP and LDP and as the reference population to predict the GEBV of the validation samples. The validation population included animals born in or after 2014 and was used for validation in the genomic prediction test

*AGE* age to 100 kg live weight, *BF* back fat thickness, *TTN* total teat number

A previous study showed that using the same reference individuals for pre-selecting variants through a GWAS as the GP training population can decrease prediction accuracy and result in biased predicted breeding values [23]. Considering this factor and to prevent overfitting, in this study, we divided the animals (after genotype quality control) into three non-overlapping populations. Matching the breeding practice, 441 pigs born in or after 2014 were treated as a validation population and were used to predict GEBV and determine accuracy and bias; 1000 individuals were randomly selected in the remaining samples as the discovery population, which were used to detect QTL or significant SNPs by GWAS; and the remaining 2108 samples were treated as a training population to predict the GEBV of the validation population. Selective linkage disequilibrium pruning and linkage disequilibrium pruning (LDP) parameters were optimized in the training population by five-fold cross-validation.

### Genotype data

The genomes of all boars were sequenced on an Illumina (paired-end 150) or BGI (paired-end 100) platform, with an average sequencing depth of ~ 0.68 × for all individuals. SNP calling for these individuals was performed following the processing procedures for LCS data as previously described [28]. The BaseVar [34] version 1.01 and STITCH [35] version 2.0 software were used to call SNP variants and impute genotypes, respectively. After SNP calling and genotype imputation, 15,689,585 autosomal SNPs were identified in the study population. SNPs with a minor allele frequency (MAF) < 0.05 and a call rate < 0.95, and individuals with a call rate < 0.90 were excluded from further analyses using the VCFtools (version 1.17). After quality control, 3549 of the 3579 pigs and 10,109,688 SNPs were retained for subsequent analyses.

SNPs on the commercial chip 80k (CC 80k), commercial chip 50k (CC 50k), and GBS panel were extracted from the LCS clean panel for marker detection simulation to evaluate the performance of different genotyping technologies in GP. Details on the GBS markers are reported in [30]. Some SNPs might be missed as LCS cannot capture all the genome variants. To ensure a more objective evaluation of the marker panels, the missing SNP data were represented by the nearest markers (within 1 kb upstream/downstream region) and only autosomal SNPs were retained. Finally, 55,216, 36,851, and 94,832 SNPs from the CC 80k, CC 50k, and GBS panels, respectively, were included in this study.

### Trait simulation

To evaluate the performance of SLDP under known genetic architectures, we conducted phenotypic simulations based on real LCS 10.1 M genotypes. In the data simulation, only additive genetic and residual effects were considered. We simulated six traits, each with different numbers of quantitative trait nucleotides (QTN) (100 or 10,000) and heritability levels (h^2^: 0.15, 0.30, or 0.50). The lower QTN condition (100) represented simulation scenarios where traits are influenced by a few major genes, while the higher QTN condition (10,000) represented scenarios of traits controlled by an infinite number of loci. We used the R package SIMER (https://github.com/xiaolei-lab/SIMER) for the simulation analyses. QTN were randomly selected from all SNPs and their effects were sampled from the same standard normal distribution using the SIMER software with default settings. Residual terms were sampled from another normal distribution with the variance parameter adjusted to the heritability level.

### GWAS for the selection of significant sequence variants in the discovery population

To obtain prior information for the GP tests, we conducted GWAS on 10,109,688 clean genotypes from the LCS panel for each real and simulated trait. Association results based on the discovery population were used for subsequent SLDP marker selection. The mixed linear model used in the GWAS and fitted using the GCTA “mlma” model (version 1.92.0) [36], was as follows:

$$\mathbf{y}=\mathbf{X}{\varvec{\upbeta}}+{\upalpha }\mathbf{b}+\mathbf{W}\mathbf{g}+\mathbf{e},$$

where $\mathbf{y}$ is the vector of phenotypes; ${\varvec{\upbeta}}$ is the vector of fixed effects, including the year and season effects; α is the effect of a candidate SNP and $\mathbf{b}$ is the corresponding vector of genotypes (coded 0, 1, and 2); $\mathbf{g}$ is the vector of random polygenic effects, with $\mathbf{g}\sim N(0,\mathbf{G}{\upsigma }_{\mathrm{g}}^{2})$; $\mathbf{G}$ is the GRM constructed using LCS SNPs; ${\upsigma }_{\mathrm{g}}^{2}$ is the variance explained by SNPs; $\mathbf{e}$ is the vector of random residual effects, with $\mathbf{e}\sim N(0,\mathbf{I}{\upsigma }_{\mathrm{e}}^{2})$; $\mathbf{I}$ is the identity matrix; and $\mathbf{X}$ and $\mathbf{W}$ are the design matrices connecting phenotypes to fixed effects and random polygenic effects, respectively.

Implementing a strict threshold may result in false negatives, while a relaxed threshold may introduce false positives. To capture more genetic effects and explore the optimal parameter combination for each trait (defined as parameters combined with the highest prediction accuracy in the training population via five-fold cross-validation for each trait), we established a relatively conservative P-value threshold gradient of 0.0001 to 0.01 to select critical markers. We defined genome regions of 50 kb on either side of each associated SNP as QTL-covered regions.

For the simulated traits, markers in high LD with QTN are likely to emerge as significant. Hence false-positive SNPs were defined as non-QTN SNPs that have a low LD (r^2^ < 0.50) with all the actual QTN. Detected QTN or markers that have a higher LD (r^2^ ≥ 0.50) with QTN were classified as informative SNPs. The estimated false-positive rate was calculated by dividing the number of false-positive sites by the number of detected sites.

### Genomic prediction models

To assess the universality of our marker selection method, we used two representative models for GP: GBLUP and BayesR [37]. We carried out a five-fold cross-validation in the training population to optimize the parameters of SLDP and LDP. The accuracy of the different models and marker panels was then evaluated in the validation population. Accuracy was defined as the Pearson correlation between GEBV and the true phenotype for the real traits, and between GEBV and the true breeding value (TBV) for the simulated traits. In addition, bias was identified as the regression coefficient of phenotypes or TBV on GEBV.

### GBLUP

Genomic estimated breeding values based on GBLUP were estimated for individuals using the following equation:

$$\mathbf{y}=\mathbf{X}{\varvec{\upbeta}}+\mathbf{Z}\mathbf{u}+\mathbf{e},$$

where $\mathbf{y}$ is the vector of phenotypes; ${\varvec{\upbeta}}$ is the vector of fixed effects, including the year and season effects, and only the overall mean was included in the simulation data; $\mathbf{u}$ is the vector of GEBV, with $\mathbf{u}\sim N(0,\mathbf{G}{\upsigma }_{\mathrm{g}}^{2})$; ${\upsigma }_{\mathrm{g}}^{2}$ is the genetic variance explained by SNPs; $\mathbf{e}$ is the vector of random residuals, with $\mathbf{e}\sim N(0,\mathbf{I}{\upsigma }_{\mathrm{e}}^{2})$; $\mathbf{G}$ is the GRM estimated using each SNP panel; $\mathbf{I}$ is the identity matrix; ${\upsigma }_{\mathrm{e}}^{2}$ is the residual variance; and $\mathbf{X}$ and $\mathbf{Z}$ are the design matrices connected to fixed effects and GEBV, respectively. Genomic relationship matrices were constructed using the GCTA software [36], while the prediction model was fitted using the MTG2 software [38].

### BayesR

The BayesR analytical methodology described by Erbe et al. [39] was used with a mixture of normal distributions as the prior for variant effects, including one distribution that sets the variant effects to zero, and the model was fitted using the following equation:

$$\mathbf{y}=\mathbf{X}{\varvec{\upbeta}}+\mathbf{Z}{\varvec{\upvarepsilon}}+\mathbf{e},$$

where $\mathbf{y}$ is the vector of phenotypes; ${\varvec{\upbeta}}$ is the vector of fixed effects, including the overall mean, year, and season effects, and only the overall mean was included in the simulation data; $\mathbf{Z}$ is the design matrix of variant genotypes; ${\varvec{\upvarepsilon}}$ is the vector of variant effects, distributed as a mixture of four normal distributions, including a null distribution, $N(0, 0{\upsigma }_{\mathrm{g}}^{2})$, and three others, $N(0, 0. 0001{\upsigma }_{\mathrm{g}}^{2})$, $N(0, 0.001{\upsigma }_{\mathrm{g}}^{2})$, and $N(0, 0.01{\upsigma }_{\mathrm{g}}^{2})$, where ${\upsigma }_{\mathrm{g}}^{2}$ is the additive genetic variance for each trait; $\mathbf{e}$ is the vector of random residuals, with $\mathbf{e}\sim N(0,\mathbf{I}{\upsigma }_{\mathrm{e}}^{2})$; $\mathbf{I}$ is the identity matrix; and ${\upsigma }_{\mathrm{e}}^{2}$ is the residual variance. Variance effects were estimated using the Markov chain Monte Carlo approach and BayesR software [37] with default settings.

### Selection of variants from LCS by LDP and SLDP

Due to the high level of LD in the genome, most SNPs are redundant; a previous study suggested that an excess of SNPs in high LD might introduce noise and cause biased GP [40]. Consequently, prediction accuracy might be improved by applying LDP. For traditional LDP, we used a sliding window-based LD pruning method, setting the window and step size to 500 and 200 SNPs, respectively. Any SNP pairwise r^2^ values equal to or greater than the threshold was removed. In LDP, we used 20 r^2^ thresholds that ranged from 0.05 to 1; for example, r^2^ = 1 meant that only SNPs in complete LD were removed, while the remaining SNP set was labeled as “LCS_LD1.”

**Fig. 1:**
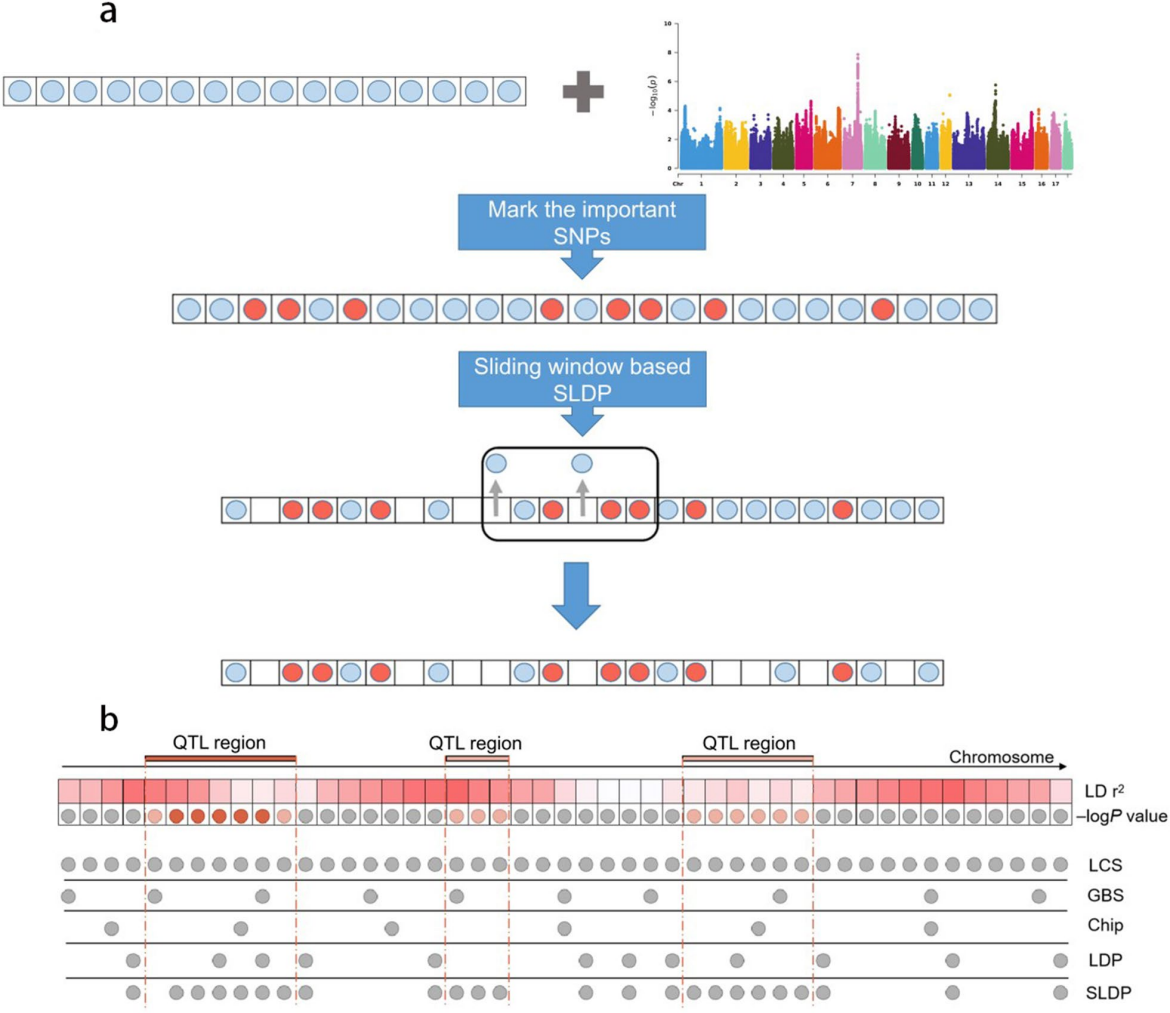
Schematic representation of SLDP and different SNP distributions across the genome. **a** Schematic representation of SLDP. SNPs (per cycle) marked in light red represent “important SNPs,” while those in light blue represent other SNPs. The set of “important SNPs” is marked according to GWAS prior information. SLDP retains all “important SNPs” in sliding window-based LD pruning. **b** Schematic illustration of the SNP distribution for different marker sets. The SNPs along the genome are colored according to their GWAS–log*P* value, with red and gray indicating high or low significance levels in GWAS, respectively. The GBS and chip sets were extracted from the LCS panel and SNPs non-overlapping with the LCS panel nearest and within the 1 kb range were extracted; only autosomal SNPs were retained

Selective linkage disequilibrium pruning considers prior information and amplifies the weights of candidate QTL regions in the prediction model. Figure 1a provides a schematic representation of SLDP. For each real and simulated trait, we first identified the “important SNPs” in the LCS panel, defined as significant SNPs with a P-value below the association threshold (gradient from 0.0001 to 0.01) or SNPs in high LD (r^2^ > 0.90) with any significant SNPs. Then, we applied the same sliding window-based LD pruning method as used in LDP; however, all “important SNPs” were retained after SLDP. We tested 180 parameter combinations (9 P-values × 20 LD r^2^ values) for each real and simulated trait. For the simulated traits, both GWAS and real QTN locations were used in SLDP (marked as SLDP_GWAS and SLDP_QTN, respectively). In SLDP_QTN scenarios, the “important SNPs” were defined as real QTN or SNPs in high LD (r^2^ > 0.90) with any real QTN. Figure 1b illustrates the difference in SNP distribution between each marker panel. After artificial modification, more SNPs were enriched in candidate QTL regions in the SLDP panel. We used the PLINK [41] software and scripts coded by the authors for these analyses.

## Results

### Phenotypic distribution and population structure

Table 1 and Additional file 1: Fig. S1 present the basic statistical characteristics of the discovery, training, and validation populations. Probably due to artificial selection, compared to the other populations, the validation population displayed phenotypic progress. AGE and BF conformed to a normal distribution, as confirmed by the Kolmogorov–Smirnov test (P = 0.326 for AGE and P = 0.235 for BF), but not TTN (P < 2.20 × 10^–16^), since it showed a discontinuous distribution. We conducted a principal component analysis (PCA) using the LCS_LD0.90 SNP set to determine the population structure of all the populations and samples. Additional file 2: Fig. S2 shows the three populations with the mixed state in principal components 1 to 3 with no sub-group stratification. This revealed the effectiveness of using marker pre-selection and prior GWAS information that was identified in the discovery population.

### Genetic parameters and architecture

Table 2 shows the results of the variance component estimates obtained through GBLUP and BayesR for each marker panel. Although there were large differences in the number of markers between the different panels, no significant difference was observed in the heritability estimated with either GBLUP or BayesR. However, the estimated heritability using the BayesR model was significantly higher than that using the GBLUP model for AGE and slightly higher for BF. For example, the estimated heritability using the CC 80k panel in the BayesR model was 0.17 for AGE and 0.36 for BF, higher than the respective estimates of 0.13 and 0.33 based on the GBLUP model. This may be attributed to the greater ability of the Bayes model than GBLUP to capture the QTL effect since only BF and AGE were subjected to artificial selection in this population.

**Table 2:** Variance components for different traits estimated on four marker panels, based on GBLUP and BayesR

Trait	Marker panel	Number of markers	Vg (se)		Ve (se)		Heritability (se)	
			GBLUP	BayesR	GBLUP	BayesR	GBLUP	BayesR
AGE	CC 80k	55,216	9.89 (2.30)	11.97 (3.27)	68.37 (2.58)	58.38 (3.22)	0.13 (0.03)	0.17 (0.03)
	CC 50k	36,851	9.87 (2.33)	10.20 (3.43)	68.51 (2.57)	59.92 (3.24)	0.13 (0.03)	0.15 (0.03)
	GBS	89,012	9.30 (2.22)	10.13 (3.29)	68.82 (2.57)	59.84 (3.46)	0.12 (0.03)	0.15 (0.04)
	LCS	10,109,688	9.92 (2.31)	11.42 (3.86)	68.32 (2.58)	58.59 (4.03)	0.13 (0.03)	0.16 (0.04)
BF	CC 80k	55,216	1.31 (0.17)	1.43 (0.17)	2.63 (0.12)	2.54 (0.13)	0.33 (0.03)	0.36 (0.03)
	CC 50k	36,851	1.29 (0.17)	1.37 (0.18)	2.67 (0.11)	2.60 (0.13)	0.33 (0.03)	0.34 (0.03)
	GBS	89,012	1.25 (0.16)	1.37 (0.17)	2.66 (0.11)	2.57 (0.13)	0.32 (0.03)	0.35 (0.03)
	LCS	10,109,688	1.28 (0.17)	1.43 (0.19)	2.64 (0.11)	2.53 (0.13)	0.33 (0.03)	0.36 (0.04)
TTN	CC 80k	55,216	0.31 (0.04)	0.29 (0.05)	0.81 (0.03)	0.81 (0.03)	0.27 (0.03)	0.26 (0.03)
	CC 50k	36,851	0.32 (0.04)	0.27 (0.05)	0.80 (0.03)	0.82 (0.03)	0.28 (0.03)	0.25 (0.04)
	GBS	89,012	0.29 (0.04)	0.29 (0.05)	0.82 (0.03)	0.80 (0.03)	0.26 (0.03)	0.26 (0.03)
	LCS	10,109,688	0.29 (0.04)	0.29 (0.05)	0.82 (0.03)	0.81 (0.04)	0.26 (0.03)	0.27 (0.04)

*AGE* age to 100 kg live weight, *BF* back fat thickness, *TTN* total teat number, *CC* 80k commercial chip 80K, *CC* commercial chips 50K, *GBS* genotyping-by-sequencing, *LCS* low-coverage sequencing

Table 3 lists the number of SNPs that were selected under varying P-value thresholds, ranging from 0.0001 to 0.01. Additional file 3: Fig. S3 presents the Manhattan plot for AGE, BF, and TTN in the discovery population, non-discovery population, and all samples. Under a strict threshold condition (P < 0.0001), a significantly larger number of SNPs was obtained for BF and TTN than for AGE, with almost no significant SNPs detected for AGE. Although numerous significant SNPs were identified for TTN, most of them (64%) were concentrated on the known *Sus scrofa* 11.1 chromosome (SSC)7 QTL region. We further analyzed the genomic regions covered by significant (P < 0.0001) SNPs for each trait. Genomic regions of 50 kb on either side of each associated SNP were considered as QTL-covered regions; the 343 SNPs for AGE spanned a 7.10-Mb genomic region, the 12,624 SNPs for BF spanned a 21.30-Mb genomic region, and the SNP number for TTN i.e., 12,343 SNPs similar to that for BF in TTN spanned only a 13.87-Mb genomic region. These results suggest that the QTL distribution varies between traits.

**Table 3:** SNPs detected using different P-value thresholds in the discovery population, the non-discovery population, and in all 3549 samples

Trait	Population	P-value thresholds								
		0.0001	0.0003	0.0005	0.0007	0.001	0.003	0.005	0.007	0.01
AGE	Discovery	105	1300	2994	5158	9052	27,662	43,604	62,631	94,385
	Non-discovery	319	1588	4281	7093	11,312	31,880	54,720	79,016	103,418
	All	343	2273	5152	6686	8537	28,082	49,399	73,167	108,976
BF	Discovery	4342	6403	7591	9236	12,033	32,926	54,589	73,302	101,580
	Non-discovery	8711	15,582	20,086	23,274	27,369	44,655	60,946	79,791	106,278
	All	12,624	16,895	19,203	21,012	23,068	40,461	59,719	77,576	105,749
TTN	Discovery	2774	7079	12,726	17,925	22,400	43,547	63,641	80,742	107,811
	Non-discovery	5733	11,605	15,370	19,011	23,697	47,462	64,705	83,287	111,884
	All	12,343	20,169	23,003	25,560	29,767	54,070	74,004	92,246	116,156

SNPs with a P-value lower than each threshold were selected for each trait. Non-discovery means the samples include 2108 training and 441 validation samples

*AGE* age to 100 kg live weight, *BF* back fat thickness, *TTN* total teat number

Subsequently, we estimated the proportion of SNPs in each distribution using the BayesR model. Overwhelmingly, most (more than 97%) SNPs had a variance of 0 for all traits and were estimated to be in the first distribution. TTN had a higher percentage of SNPs with a variance of 0 (98.17%) and a larger proportion of SNPs in the fourth distribution (0.01 * σ^2^_g_) than AGE and BF. Conversely, BF had more SNPs with a variance different from 0 and the highest proportion of SNPs in the second and third distributions yet the smallest number of SNPs in the fourth distribution (Table 4). These results confirmed that fewer QTL control TTN, while BF and AGE are influenced by a larger number of QTL with a small effect. Moreover, the detection of any definite QTL associated with AGE in this study proved challenging.

**Table 4:** Proportion of the number of SNPs and genetic variance explained in the four normal distributions modeled in BayesR

Trait	0 * ${{\varvec{\upsigma}}}_{\mathbf{g}}^{2}$		0.0001 * ${{\varvec{\upsigma}}}_{\mathbf{g}}^{2}$		0.001 * ${{\varvec{\upsigma}}}_{\mathbf{g}}^{2}$		0.01 * ${{\varvec{\upsigma}}}_{\mathbf{g}}^{2}$	
	P_num_ (%)	P_var_ (%)	P_num_ (%)	P_var_ (%)	P_num_ (%)	P_var_ (%)	P_num_ (%)	P_var_ (%)
AGE	97.42	0	2.30	29.17	0.24	30.34	0.032	40.20
BF	97.29	0	2.37	29.85	0.32	39.03	0.028	30.89
TTN	98.17	0	1.52	19.21	0.26	32.86	0.040	47.64

Results were obtained using the LCS_LD0.90 marker set

*σ*^***2***^_***g***_ genetic variance explained by the LCS_LD0.90 marker set, *P*_*num*_ proportion of the number of SNPs in a distribution to the total SNP set (LCS_LD0.90), *P*_*var*_ proportion of genetic variance explaining ${\upsigma }_{\mathrm{g}}^{2}$, *AGE* age to 100 kg live weight, *BF* back fat thickness, *TTN* total teat number

### Genomic prediction based on different marker density panels using real data

We used two SNP chips and two sequencing-based methods (GBS and LCS) for GP in order to investigate the effects of marker density and SNP genotyping on prediction accuracy and bias. Marker density exhibited a several 100-fold increase from the SNP chip to LCS data (Table 2). However, an increase in marker density did not correspondingly improve prediction accuracy; in some cases, the accuracy even decreased to a certain extent when using the GBLUP model (Table 5). Apart from the LCS_LD1 set, the CC 80k panel was particularly effective for GP of AGE, demonstrating a 2.60% higher accuracy than the LCS panel. GBS outperformed the other panels in terms of accuracy for BF and TTN. Table 6 provides the prediction accuracy and bias for the three traits based on the BayesR model; the LCS panel was not included in this analysis due to the extensive computational resources necessary for BayesR with over 10 million SNPs. The LCS_LD1 set showed a significant advantage for AGE and BF in both GBLUP and BayesR models. In addition, the accuracy of BayesR was superior to that of GBLUP for all three traits across all marker panels, exhibiting a 5.4 to 7.3% increase for AGE, a 0.2 to 2.6% increase for BF, and a 1.7 to 2.5% increase for TTN. The bias due to using the LCS_LD1, CC 80k, and LCS panels in the GBLUP model, and that due to using the CC 80k, LCS_LD1, and CC 50k panels in the BayesR model, were relatively lower than those due to using other SNP panels for the three real traits. Compared to the two SNP chips, the use of unfiltered WGS data did not improve the prediction accuracy in the GBLUP model. However, prediction accuracy increased when SNPs in perfect LD were removed from the LCS panel for AGE and BF. This result indicated that WGS data does not offer accuracy and bias advantages over the SNP array or GBS when all markers are used.

**Table 5:** Prediction accuracy and bias in the validation population for three traits based on GBLUP

	CC 50k		CC 80k		GBS		LCS_LD1		LCS	
	Accuracy	Bias	Accuracy	Bias	Accuracy	Bias	Accuracy	Bias	Accuracy	Bias
AGE	0.219	1.213	0.229	1.285	0.196	1.108	0.239	1.016	0.203	1.104
BF	0.375	1.020	0.363	1.003	0.377	1.137	0.381	1.157	0.376	1.120
TTN	0.344	0.955	0.346	0.933	0.356	0.982	0.345	0.973	0.351	0.993

Accuracy was defined as the Pearson correlation between the GEBV and phenotypes, while bias was defined as the regression coefficient of phenotypes on GEBV

*AGE* age to 100 kg live weight, *BF* back fat thickness, *TTN* total teat number, *CC 80 k* commercial chip 80k, *CC 50k* commercial chip 50k, *GBS* genotyping-by-sequencing, *LCS* low-coverage sequencing

**Table 6:** Prediction accuracy and bias in the validation population for three traits based on BayesR

	CC 50k		CC 80k		GBS		LCS_LD1	
	Accuracy	Bias	Accuracy	Bias	Accuracy	Bias	Accuracy	Bias
AGE	0.280	0.847	0.283	0.893	0.269	0.861	0.293	0.882
BF	0.379	0.860	0.389	0.949	0.379	0.885	0.401	1.025
TTN	0.365	0.880	0.363	0.851	0.375	0.884	0.370	0.872

Accuracy was defined as the Pearson correlation between the: GEBV) and phenotypes, while bias was defined as the regression coefficient of phenotypes on GEBV

*AGE* age to 100 kg live weight, *BF* back fat thickness, *TTN* total teat number, CC 80k commercial chip 80k, *CC 50k* commercial chip 50k, *GBS* genotyping-by-sequencing, *LCS_LD1* low-coverage sequencing set after removing SNPs in complete LD with other SNPs

### Prediction performance of the top 10% and bottom 10% SNPs in GWAS

**Fig. 2:**
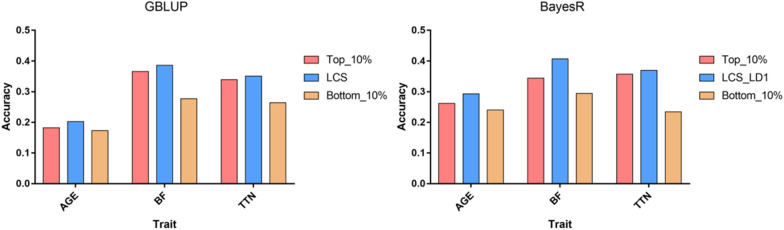
Comparison of genomic prediction accuracy. Comparison of genomic prediction accuracy of the top 10%, LCS or LCS_LD1, and bottom 10% SNPs in the GBLUP and BayesR models in the validation population. *AGE* age to 100 kg live weight, *BF* back fat thickness, *TTN* total teat number, *Top_10%* the top 10% significant SNPs in the discovery GWAS, *LCS* low-coverage sequencing, *Bottom_10%* the bottom 10% significant SNPs in the discovery GWAS

SNPs in the LCS panel were ranked according to their GWAS P-values for the three real traits in the discovery population (from small to large). The top 10% and bottom 10% SNPs were selected for accuracy assessment in the validation population to explore the performance of phenotypically related and unrelated SNPs during GP. The prediction accuracy of the top 10% SNPs for each trait was significantly higher than that of the bottom 10% SNPs in both the GBLUP and BayesR models (Fig. 2). This indicates that the contribution of markers to the prediction differed throughout the genome and suggested that markers exhibiting a higher correlation with QTL had higher prediction power than the others. Both informative and noisy markers exist in WGS data, which may explain why the WGS data did not have a significant advantage in GP compared with SNP chips. This also suggested that pre-selecting SNPs from high-density WGS panels may improve the prediction accuracy.

### Prediction performance of LDP and SLDP

**Fig. 3:**
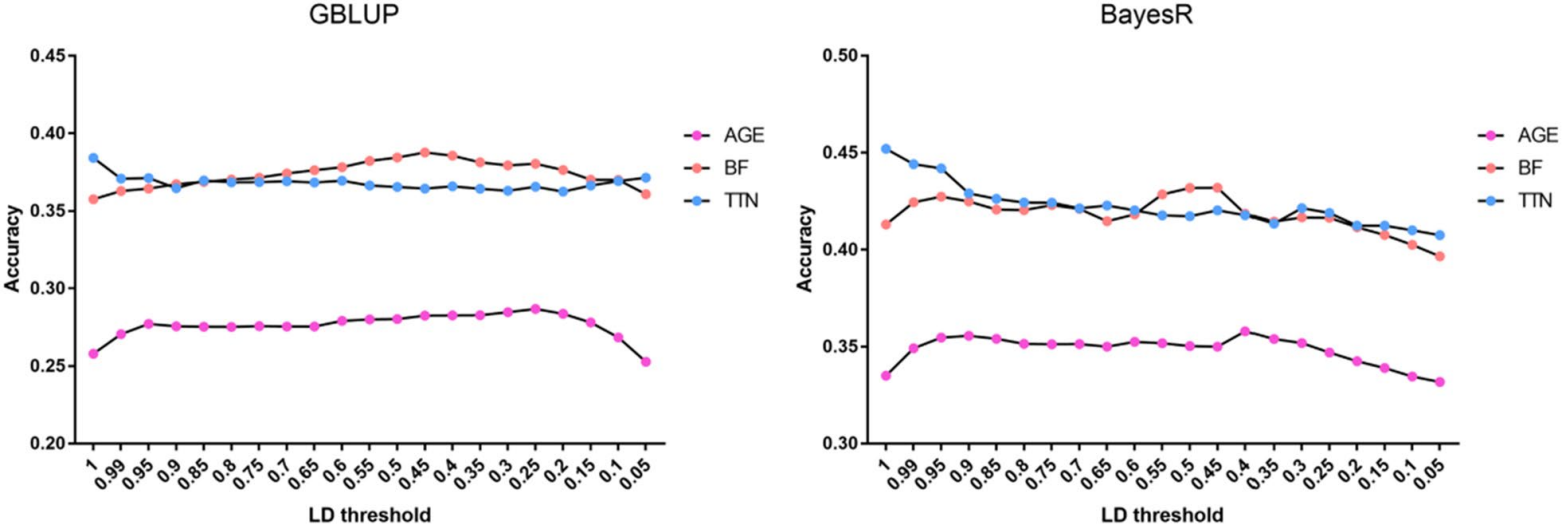
Genomic prediction accuracy of the LCS panel after LDP. Genomic prediction accuracy of the LCS panel after LDP according to the r^2^ gradient (from 1 to 0.05) in the GBLUP and BayesR models in the training population. *AGE* age to 100 kg live weight, *BF* back fat thickness, *TTN* total teat number. The accuracy was obtained by five-fold cross-validation in the training population and defined as the Pearson correlation between GEBV and phenotypes

In this study, the LCS panel was filtered using a sliding-window LDP approach based on r^2^ threshold gradients (see “Methods”); a lower r^2^ indicated that fewer markers remained. Additional file 4: Table S1 shows the remaining number of SNPs after filtering with different thresholds. The number of LCS SNPs decreased rapidly after LD filtering. For example, when r^2^ was > 0.99, the number of LCS SNPs decreased from 10.1 M to 361 K. The accuracy of the GBLUP and BayesR models for predicting the three real traits after LDP exhibited different patterns, as shown in Fig. 3. In the GBLUP model, with a decrease in r^2^, the accuracy of GP for AGE and BF initially increased and then decreased; by contrast, the accuracy of GP for TTN decreased with a lower r^2^ setting. In the BayesR model, the general trend was the same as that found in GBLUP. In contrast, for TTN, the accuracy of the BayesR model declined faster than that of GBLUP, with a decrease in r^2^.

**Fig. 4:**
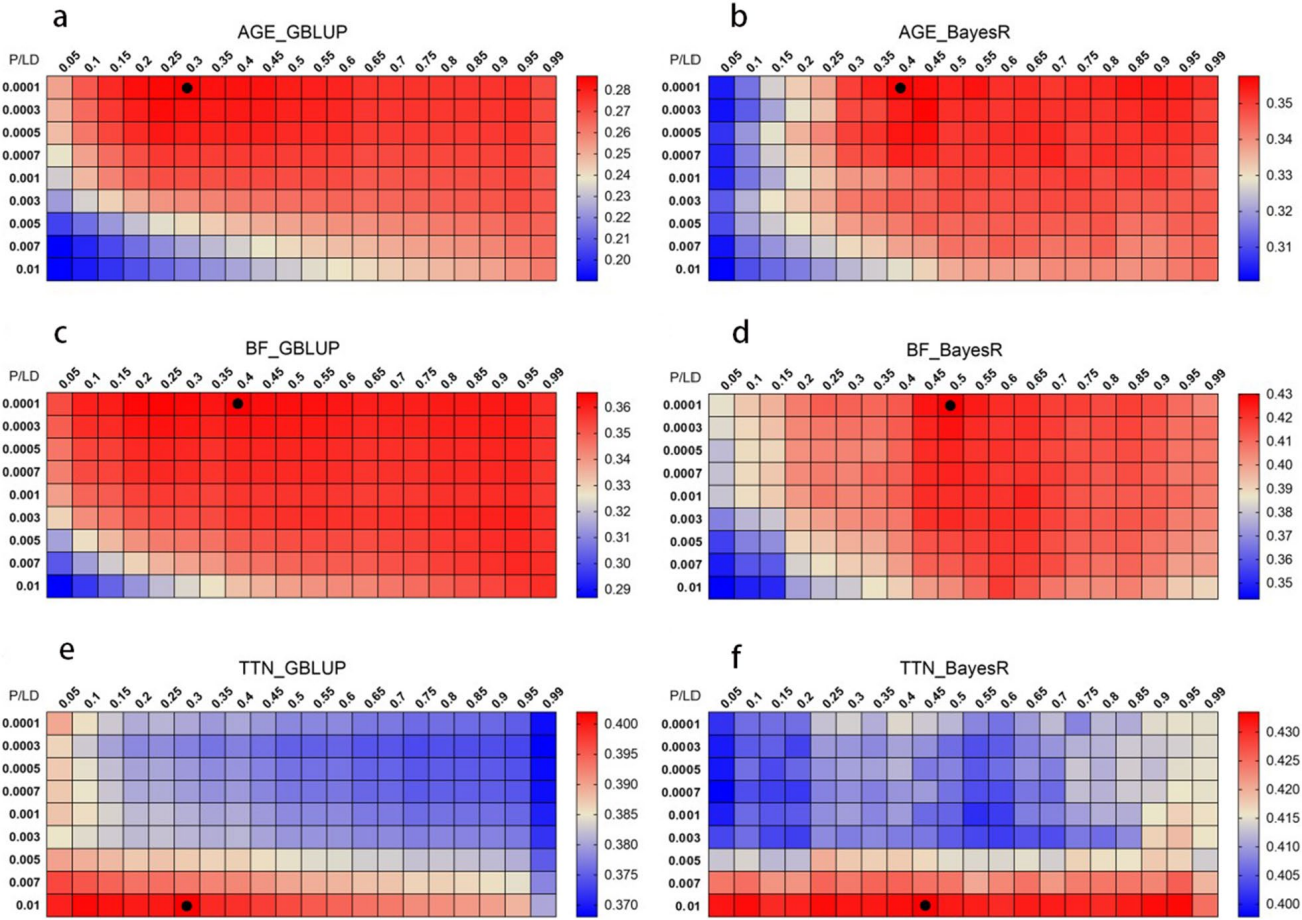
Heat map of genomic prediction accuracy for three real traits using SNPs after SLDP. Heat map of genomic prediction accuracy for three real traits using SNPs after SLDP according to the r^2^ (from 1 to 0.05) and P-value (from 0.0001 to 0.01) gradient in the training population. AGE, age to 100 kg live weight; BF, back fat thickness; TTN, total teat number. Panels **a**, **c**, and **e** show the results of the GBLUP model; panels **b**, **d**, and **f** show the results for the BayesR model. Each square represents a parameter combination and the accuracies are indicated with deeper colors corresponding to high (red) or low (blue) accuracy. Black dots mark the optimal combination of parameters for accuracy. The accuracy was obtained by five-fold cross-validation in the training population and defined as the Pearson correlation between GEBV and phenotypes

To consider GWAS prior information in GP, we used an improved LDP method termed SLDP. Figure 4 shows the prediction accuracy of the 180 combinations of parameters for the three real traits in the GBLUP and BayesR models used on the training population determined by five-fold cross-validation. The number of SNPs used in each scenario for AGE, BF, and TTN is shown in Additional files 5, 6, 7: Tables S2, S3, S4, respectively. For the GBLUP model, the accuracy of prediction for AGE and BF was more affected by LDP, whereas the accuracy for TN was significantly affected by the P-value. For GBLUP, the optimal parameter combinations for the GP of AGE was a P-value of 0.0001 and r^2^ of 0.30, of BF was a P-value of 0.0001 and r^2^ of 0.40, and of TN was a P-value of 0.01 and r^2^ of 0.30 (Fig. 4a, c, and e). A similar pattern was obtained with the BayesR model, and the optimal combination of the two models had the same P-value. For BayesR, the optimal parameter combination for the GP of AGE was a P-value of 0.0001 and r^2^ of 0.40, of BF was a P-value of 0.0001 and r^2^ of 0.50, and of TN was a P-value of 0.01 and r^2^ od 0.45 (Fig. 4b, d, and f). Notably, the optimal combination for BayesR tended to have a higher r^2^ threshold, suggesting that BayesR is more suitable for high-density marker panels than GBLUP. The parameter-optimized marker sets by LDP and SLDP (i.e., those with the highest prediction accuracies for each trait) were further evaluated in the validation population. The distribution of MAF changed after LDP, with the proportion of the lower and higher MAF SNPs increasing, especially when using a lower r^2^ threshold (see Additional file 8: Fig. S4), which is a concern. Based on LDP, SLDP increases the number of SNPs in some MAF intervals since it tends to select clusters of SNPs in the genome.

**Fig. 5:**
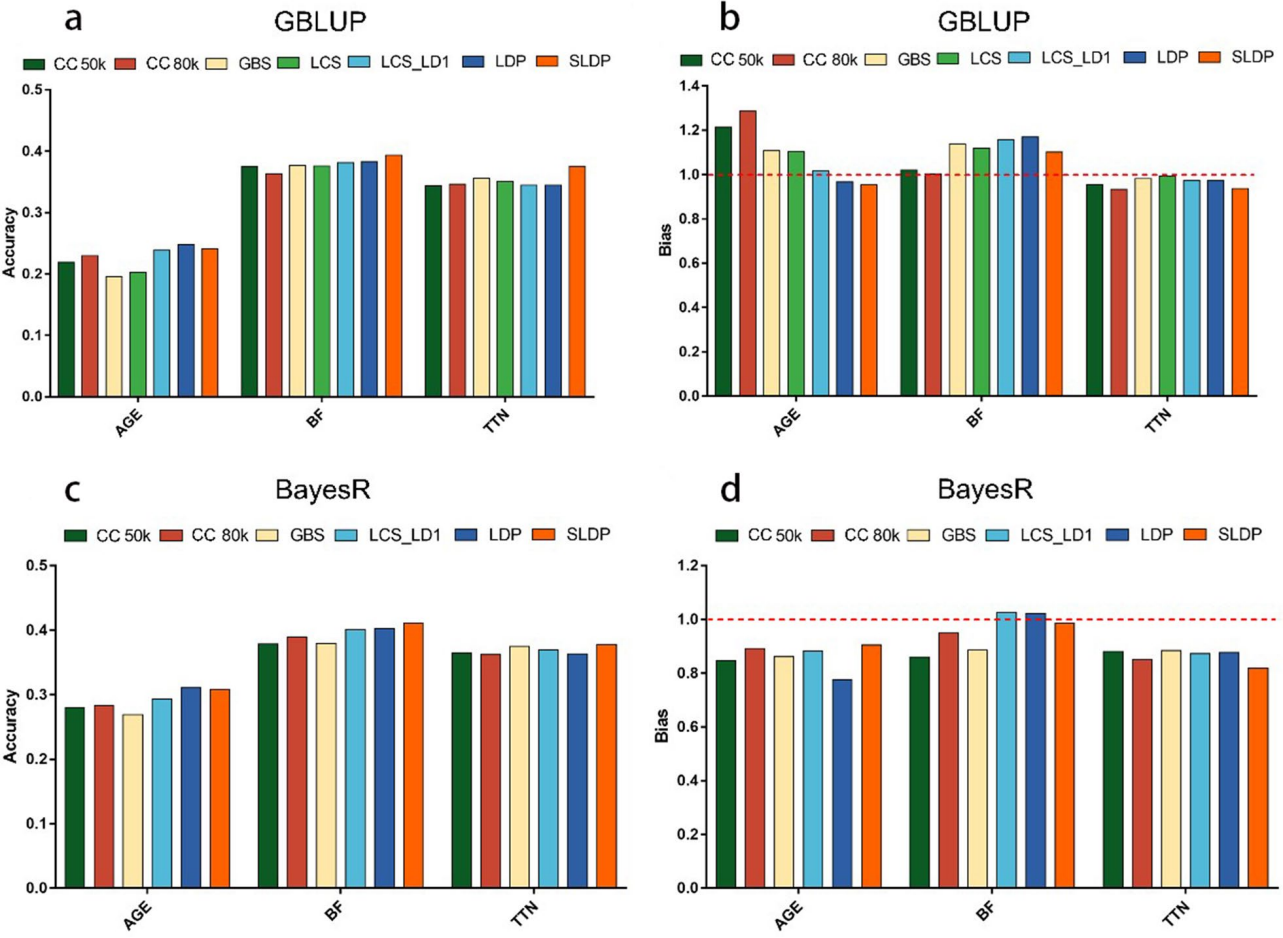
Prediction accuracy and bias of SNP sets for real traits in GBLUP and BayesR models in the validation population. **a** and **b** Results of the GBLUP model. **c** and **d** results of the BayesR model. *CC_50k* commercial chip 50K, *CC_80k* commercial chip 80K, *GBS* genotyping-by-sequencing, *LCS* low-coverage sequencing, *LCS_LD1* low-coverage sequencing set after removing completely linked disequilibrium SNPs, *LDP* linkage disequilibrium pruning and parameter (r^2^) optimized in the training population, *SLDP* selective linkage disequilibrium pruning and parameters (P-value and r^2^) optimized in the training population. The accuracy was defined as the Pearson correlation between GEBV and phenotypes

Figure 5 summarizes the accuracy of each SNP panel in the validation population. LDP had an advantage over the two SNP chips for AGE and BF, but the accuracy of LDP was relatively lower for TTN. In the GBLUP model, the accuracy of prediction based on SLDP for the three traits (AGE, BF, and TTN) increased by 2.23, 1.87, and 3.22% compared to the CC 50k chip, and increased by 1.19, 3.01, and 2.94% compared to the CC 80k chip, respectively. We also observed a similar improved accuracy for SLDP in the Bayesian model. Compared to LDP, SLDP showed an improvement of 1.04 and 3.07% in GBLUP and of 0.84 and 1.40% in BayesR for BF and TTN, respectively, but exhibited a slightly reduced accuracy for AGE. However, the optimal marker panel for bias was not robust, and SLDP did not have a significant advantage over the other panels. In summary, after marker selection, LCS data had the highest accuracy (compared to SNP chips and GBS) in most cases, indicating that using sequencing data has potential in GP.

### Prediction performance of SLDP with simulation data

**Fig. 6:**
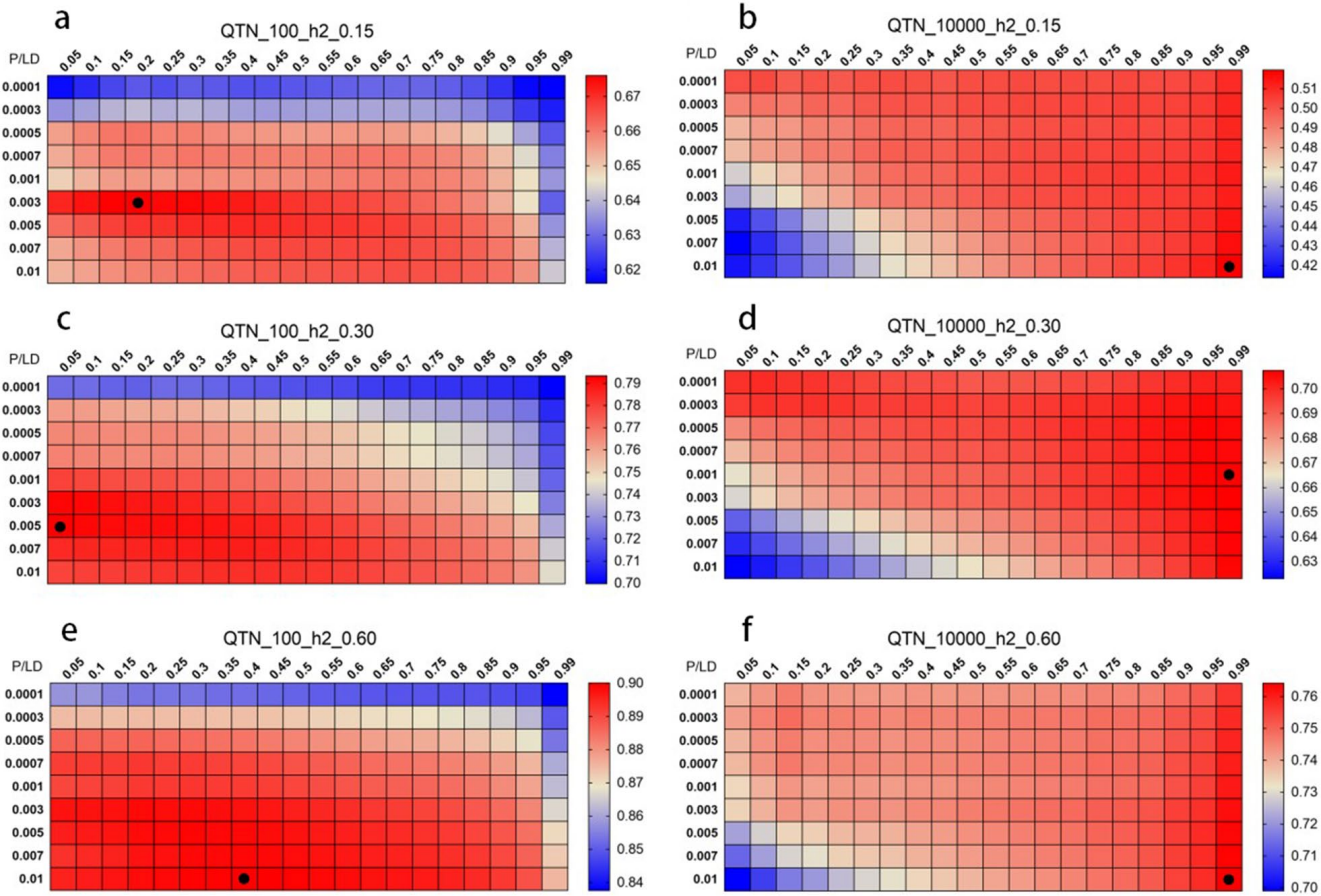
Heat map of genomic prediction accuracy for simulated traits using SNPs after SLDP. Heat map of genomic prediction accuracy for simulated traits using SNPs after SLDP according to the r^2^ (from 1 to 0.05) and P-value (from 0.0001 to 0.01) gradient in the training population. The results of the GBLUP model are shown; each square represents a parameter combination and the accuracies are indicated with deeper colors corresponding to high (red) or low (blue) accuracy. Black dots mark the optimal combination of parameters for accuracy. The accuracy was obtained by five-fold cross-validation in the training population and defined as the Pearson correlation between GEBV and TBV. **a** *QTN_100_h2_0.15* simulated traits with 100 QTN and a heritability of 0.15, **b** *QTN_10000_h2_0.15* simulated traits with 10,000 QTN and a heritability of 0.15, **c** *QTN_100_h2_0.30* simulated traits with 100 QTN and a heritability of .030, **d** *QTN_10000_h2_0.30* simulated traits with 10,000 QTN and a heritability of 0.30, **e** *QTN_100_h2_0.60* simulated traits with 100 QTN and a heritability of 0.60, **f** *QTN_10000_h2_0.60* simulated traits with 10,000 QTN and a heritability of 0.60

**Fig. 7:**
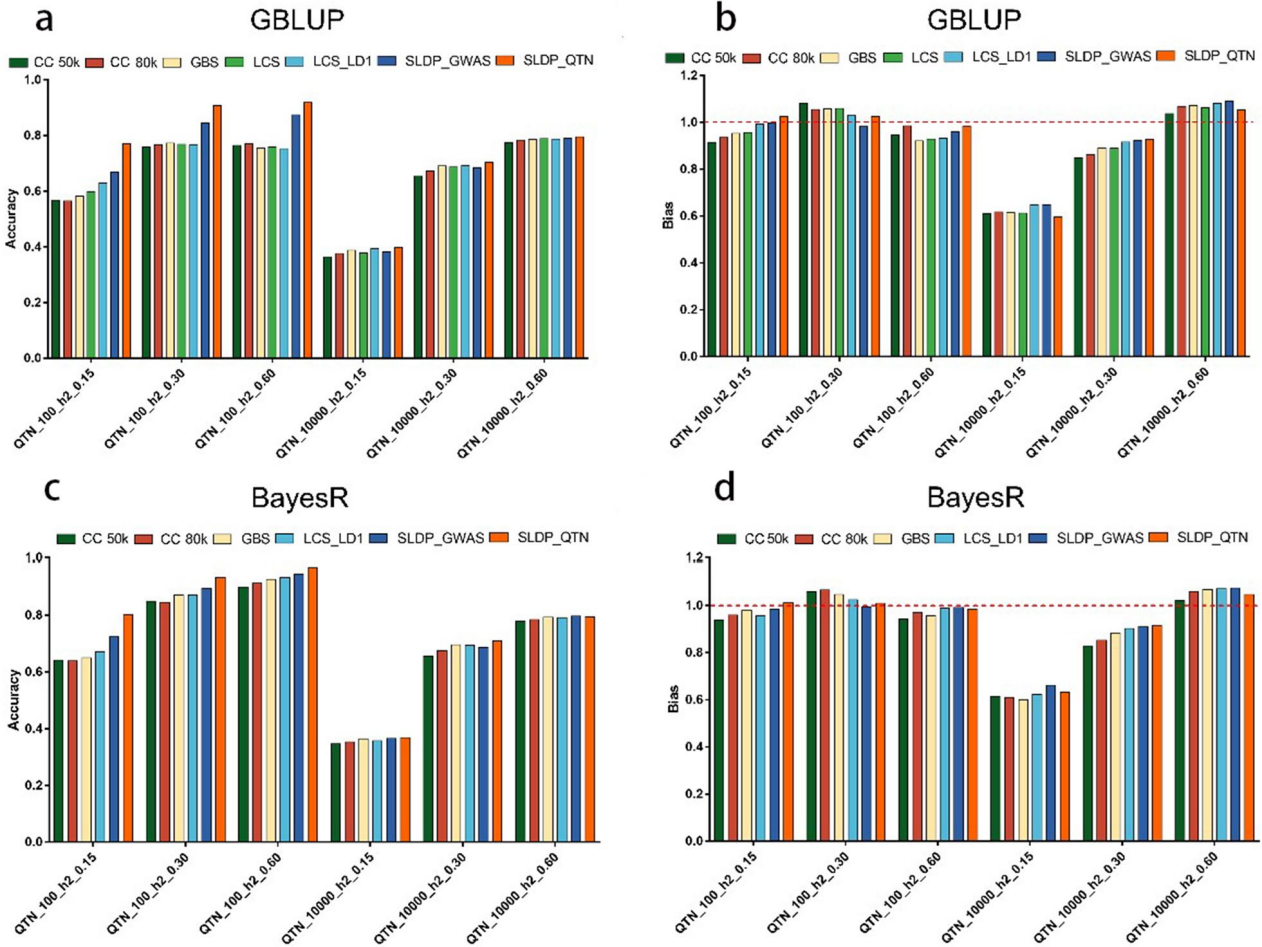
Prediction accuracy and bias of SNP sets based on simulation data with the GBLUP and BayesR models. **a** and **b** Results of the GBLUP model. **c** and **d** Results of the BayesR model. *CC_50k* commercial chip 50 K, *CC_80k* commercial chip 80 K, *GBS* genotyping-by-sequencing, *LCS* low-coverage sequencing, *LCS_LD1* low-coverage sequencing set after removing completely linked disequilibrium SNPs, *SLDP_GWAS* selective linkage disequilibrium pruning based on discovery GWAS prior and parameters (P-value and r^2^) optimized in the training population, *SLDP_QTN* selective linkage disequilibrium pruning based on real QTN. The accuracy was defined as the Pearson correlation between GEBV and TBV

To assess the performance of SLDP for evaluating different traits with a known genetic structure, we performed prediction using simulated data (see Methods). Figure 6 shows the prediction accuracy for SLDP with 180 combinations of parameters in the training population by GBLUP; the result using the BayesR model is shown in Additional file 9: Fig. S5. The traits that were controlled by fewer QTN tended to gain more accuracy improvement from GWAS prior information (with a lower LD r^2^). Figure 7 shows the prediction accuracy and bias for different SNP sets in the GBLUP and BayesR models under six simulation scenarios in the validation population. In general, the prediction accuracy of each marker panel was significantly improved with increasing simulated heritability. In the three simulation scenarios with fewer QTN, the accuracy of SLDP_QTN, where the real QTN location was used, was significantly higher than that of the other marker panels; for example, the accuracy of SLDP_QTN increased by 17.15, 13.89, and 15.92% relative to those of LCS in the GBLUP model and increased by 13.01, 6.28, and 3.39% relative to those of LCS_LD1 in the BayesR model at heritability levels of 0.15, 0.30, and 0.60, respectively. The improvement in accuracy with SLDP was clearly greater for the GBLUP model than for the BayesR model. For the scenarios using the infinitesimal model, SLDP_QTN showed only a 0 to 0.4% increase in accuracy relative to LCS in GBLUP and an increase of 0 to 0.8% in accuracy relative to LCS_LD1 in BayesR when the simulated number of QTN was 10,000.

In practice, the information on the genomic localization of all the real QTN cannot be accurately obtained; therefore, we performed SLDP based on the GWAS results where the parameters were optimized in the training population to match the real traits. For SLDP_GWAS, the accuracy also increased compared with that obtained with the other marker panels for the three traits controlled by fewer QTN. For example, the accuracy of SLDP_GWAS increased by 6.93, 7.70, and 11.47% relative to LCS in the GBLUP model and increased by 5.30, 2.45, and 1.23% relative to the LCS_LD1 set in the BayesR model at heritability levels of 0.15, 0.30, and 0.60, respectively. However, the accuracy of SLDP_GWAS was significantly lower than that of SLDP_QTN due to the presence of false positives and negatives obtained in the GWAS; that is, some noisy SNPs might have been detected, while some true QTN might have been missed. Regarding bias, both SLDP_GWAS and SLDP_QTN had no stable advantage for all traits.

**Fig. 8:**
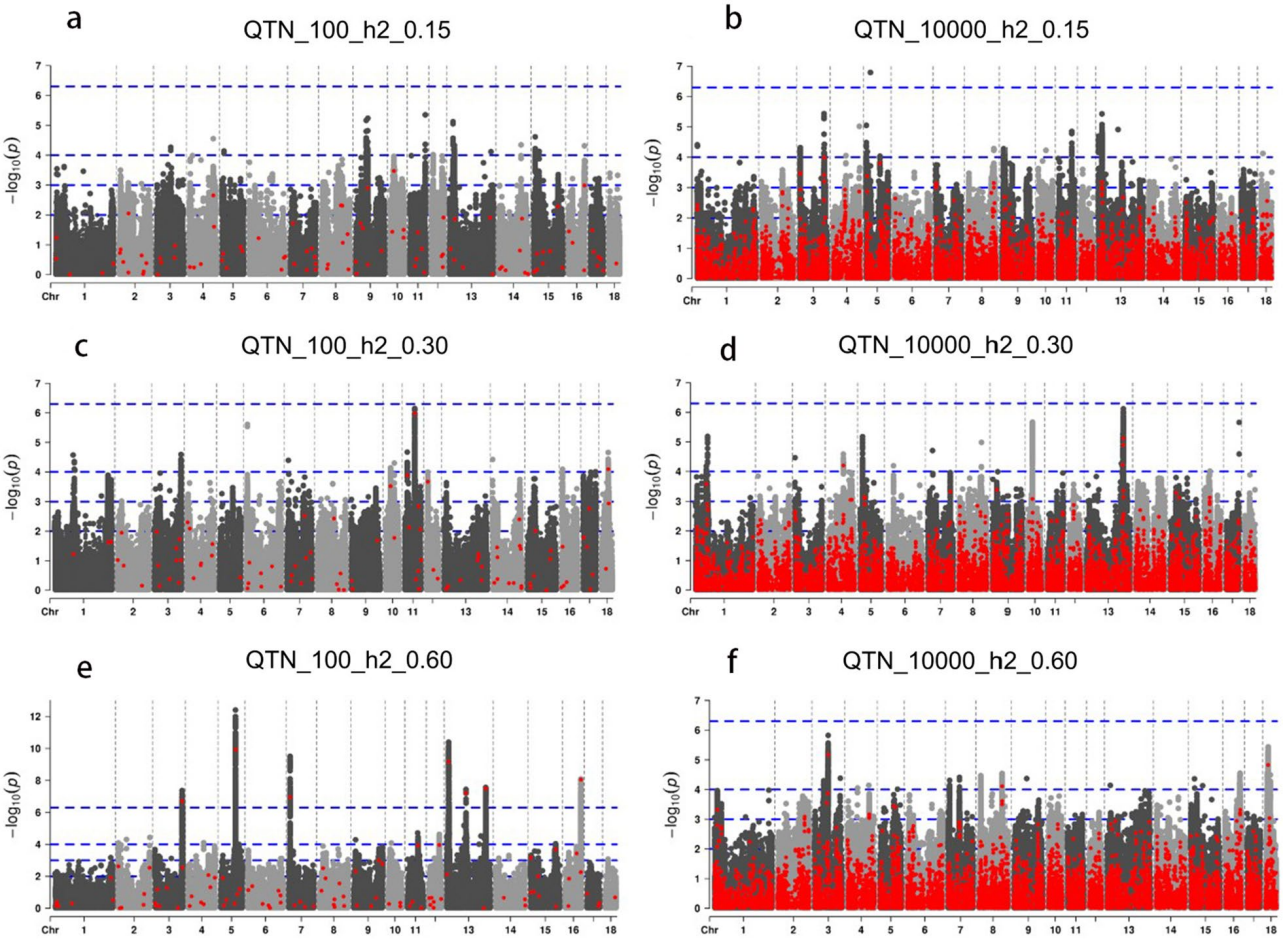
Manhattan plot of genome-wide association analysis for six simulated traits in the discovery population. Simulated QTN and other SNPs are colored by red and gray points, respectively. The blue dotted line represents the P-value threshold 5 × 10^–8^, 10^–4^, 0.001, 0.01 from top to bottom. The number of QTN detected and the estimated false-positive rate for each P-value threshold are shown in Additional file 10: Table S5. **a** *QTN_100_h2_0.15* simulated traits with 100 QTN and a heritability of 0.15, **b** *QTN_10000_h2_0.15* simulated traits with 10,000 QTN and a heritability of 0.15, **c** *QTN_100_h2_0.30* simulated traits with 100 QTN and a heritability of 0.30, **d** *QTN_10000_h2_0.30* simulated traits with 10,000 QTN and a heritability of 0.30, **e** *QTN_100_h2_0.60* simulated traits with 100 QTN and a heritability of 0.60, **f** *QTN_10000_h2_0.60* simulated traits with 10,000 QTN and a heritability of 0.60. The result shows the difficulty in detecting most QTN by GWAS, especially for the low-heritability trait

The results of the simulated GWAS based on six simulated traits in the discovery population are shown in Fig. 8 and Additional file 10: Table S5. They show that only a small proportion of QTN could be detected by GWAS, especially in the low-heritability scenarios when only 1000 samples were used. For example, only 22 of the 100 QTN could be detected at a P-value threshold of 0.05 in scenario “QTN_100_h2_0.15” (see Additional file 10: Table S5), with a 98.10% estimated false-positive rate. This indicates that the detection power of GWAS is limited. In addition, the improvement in accuracy obtained with SLDP was lower for real traits than for the simulated traits, possibly due to their simpler genetic architecture because only additive effects are considered in the simulated data and the reliable prior information obtained by GWAS for real traits is more limited. This suggests that future studies in GP should integrate more clues, in addition to those obtained from GWAS.

## Discussion

With the advent of next-generation sequencing technologies and the continuous reduction in sequencing costs, it is now feasible to apply GP using WGS data. Nevertheless, maximizing the benefits of WGS in GP remains challenging, especially when dealing with various complex traits. In this study, we systematically evaluated the factors that influence prediction accuracy when using WGS data by examining real and simulated traits in a single pig population and developed a methodology (SLDP) from the perspective of marker selection. This methodology was then used to select SNPs from a WGS high-density marker set by GWAS prior information, which led to an increase in prediction accuracy for real traits with major QTL and for simulation traits controlled by a small number of QTN. Prediction accuracy improved with the SNPs detected after SLDP compared to that obtained using data from SNP chips or GBS, suggesting that the use of pre-selected WGS data can improve GP accuracy of major traits.

The application of WGS data in GP remains contentious. Meuwissen and Goddard [25] reported that using WGS data increased the prediction accuracy of genetic values by more than 40% relative to that achieved by using a 30 k SNP chip, according to simulated data. Other simulation studies have also indicated that the use of WGS data can increase prediction accuracy [19, 20, 42]. Although the use of whole-genome data has enabled the identification of mutations underlying complex traits, the impact on GP accuracy was limited [22, 24, 43, 44]. A similar conclusion was drawn from the current study when WGS data were initially used directly, although the marker density increased by several hundred folds compared to the two SNP chips or the LCS data. The inconsistency between the results from theoretical and practical research can be attributed to two main reasons. First, van Binsbergen et al. [26] reported that, at the technical level, potential genotyping errors in a SNP array-based imputation panel might affect prediction accuracy. Our previous study [28] demonstrated that using genotypes based on LCS data, where more than 99% of the genotypes were consistent with those from the CC 80 k SNP array for common variants (MAF > 0.01), yielded higher prediction accuracy than the array-based imputation strategy [45, 46]. Therefore, we believe that genotyping errors may not be the main reason for the absence of improvement in accuracy in this study. Another hypothesis is that WGS data include many noisy sites that are useless in GP (i.e., a significant number of SNPs cannot capture the QTL effect), which may adversely affect GP [43]. To verify this hypothesis, we selected the top and bottom 10% of the GWAS SNPs in the discovery population to evaluate the accuracy of GP for three real traits. Our results suggest that the contribution of prediction accuracy varies at different loci throughout the genome, where SNPs in high LD with QTL (top SNPs) exhibit a superior predictive performance than those with unrelated markers (bottom SNPs). This finding implies that although WGS data may contain more useful information than a SNP array, this positive effect is counteracted by the introduction of noisy sites in WGS, leading to an overall limited improvement or even decrease in GP. We also investigated the effect of a range of LD pruning parameters on the GP of different traits and demonstrated that the prediction accuracy could be improved using the LD pruning method for predictions of AGE and BF. In conclusion, retaining effective sites using pre-selected markers and eliminating noisy sites could improve the accuracy of GP using WGS data.

The basic LD pruning method can remove collinear SNPs, as high LD can cause redundancy of genomic information between markers. Previous findings have suggested that LD pruning can improve prediction accuracy in WGS data [40]. Considering the advantages of using the LD pruning strategy and introducing GWAS prior information for GP in terms of accuracy and computational complexity, in this study, we combined these approaches and used a marker selection method, SLDP, to consider the prior information of GWAS during SNP filtering. Post SLDP, the marker density increased in the potential QTL region and the weight of this region increased in GP (Fig. 1). The principle is similar to that of other methods which integrate the GWAS prior into prediction models. The greatest difference with the SLDP method is that only SNP combinations are changed without model modifications, which is more convenient in real breeding practices. Further study is needed to establish the benchmark of similar methods in larger and broader data. When selecting “important SNPs,” we did not use only the significant loci (after multiple testing corrections) in this study as prior information for GWAS, since this method alone cannot confirm the true causative mutation. In contrast, multiple mutations may cumulatively contribute to a QTL, as a so-called local minor-effect polygene interaction [47]. In addition, unlike a QTL mapping study, the objective of SLDP is to enrich the predictive SNPs in WGS data rather than to define one or several causative mutations; consequently, SLDP has a higher tolerance for false positives than QTL mapping. Thus, a relatively conservative gradient P-value threshold, ranging from 0.0001 to 0.01, was used in our analyses. We used three real and six simulated traits to test the prediction accuracy and bias for SLDP; several SNP panels, including two commercial SNP chips, GBS, LCS, and basic LDP, were set as benchmarks. The parameters of SLDP were optimized in the training population by five fold cross-validation and then used for prediction in the validation population. Prediction accuracy increased for BF and TTN when SLDP was used for the three real traits compared with the accuracy obtained with the other control panels.

Moreover, using SLDP, the accuracy increased to a greater extent for TTN than for BF, as more significant and major QTL exist for TTN (see Additional file 3: Fig. S3). In contrast, accuracy of GP slightly declined for AGE when SLDP was used compared to LDP, due to the low power of the GWAS for AGE which detected only a small number of significant SNPs. Although a similar trend was observed, BayesR seemed to perform better in terms of prediction accuracy compared to GBLUP for all real traits. We believe that the main reason for the difference in prediction performance of SLDP can be primarily attributed to the substantial differences in GWAS results caused by variations in the genetic architecture of the three traits. Therefore, for further comparison, we performed analyses of the genetic architecture of the three traits. First, we estimated the heritability of all traits using GBLUP and BayesR Gibbs sampling, which showed that AGE is a complex trait with a low heritability, whereas BF and TTN have a moderate heritability. For both AGE and BF, the estimated heritability was higher when using the BayesR model than the GBLUP model, which reflects the superior accuracy of the Bayes model in evaluating genetic variance based on a higher density of markers for minor-effect polygenic traits, leading to overall higher prediction accuracy. The heritability estimates obtained for AGE and BF in this study are significantly lower than those reported in previous studies [48, 49], since the detection power of QTL decreases due to a reduction in allele frequency. The comparison of the GWAS for the three traits showed that more significant SNPs were obtained for BF and TTN than for AGE. However, the identification of significant SNPs for AGE was challenging, TTN is mainly controlled by major-effect QTL (Table 3 and see Additional file 3: Fig. S3).

Furthermore, the BayesR mixed distribution model was used to estimate the proportion of SNPs in each distribution. The results showed that TTN had the largest number of ineffective QTL and large-effect QTL, whereas BF had more QTL with a non-zero-variance and the highest proportion of QTL in the second and third distributions but relatively the lowest proportion of QTL in the fourth distribution. These results confirm that a few major QTL control TTN, whereas BF and AGE are controlled by more small-effect QTL with major and moderate QTL also existing for BF.

To further explore the influence of the genetic architecture of traits on the predictive performance of SLDP, we simulated six traits with three heritability levels controlled by 100 or 10,000 QTN. Similar to the analyses with real traits, we also used the discovery population for GWAS, the reference population for parameter optimization of SLDP, and the validation population for the prediction test. SLDP showed a significant increase in prediction accuracy for the three simulated traits with a small number of QTN; however, SLDP exhibited little to no accuracy improvement under the infinitesimal simulation scenarios. SLDP improved accuracy of prediction to a greater degree when the real positions of the QTN were used (SLDP_QTN) compared with GWAS prior-based SLDP (SLDP_GWAS) since both false positives and negatives exist in GWAS data (Fig. 8 and see Additional file 10: Table S5). SLDP had a lower bias in the three traits with fewer QTN but no advantage under infinitesimal simulation scenarios. Therefore, the results obtained for both the real and simulated traits showed that the performance of SLDP was strongly influenced by the genetic architecture of the traits and that traits with major QTN or those that are controlled by a small number of QTN were more likely to show greater improvement in accuracy. This is consistent with our expectations and previous GWAS prior-based prediction analyses such as TABLUP [13], GFBLUP [50], and KAML [14] or when combining significant SNPs from WGS with chip data [22, 43]. Previous simulation studies also reached similar conclusions [24, 51]. Two main reasons may explain this phenomenon. On the one hand, it is easier to detect significant markers in GWAS for traits that are controlled by major and fewer QTN, when sample size is limited, resulting in a larger signal-to-noise ratio of GWAS prior information and better prediction performance in SLDP. On the other hand, traits not controlled by major QTN hinder the detection of significant SNPs without significant accuracy improvement, such as the case for AGE and 10,000 QTN simulation traits in this study. Our results of the simulated GWAS also showed that, in the scenarios with traits controlled by 100 QTN, a higher proportion of real QTN was detected than in the scenarios with traits controlled by 10,000 QTN. Moreover, improving prediction accuracy by highlighting a small proportion of the SNPs or genome regions is difficult if the effects of the trait are spread across multiple variants throughout the genome. Otherwise, when too many markers or regions are weighted, the per-variant or region weight will decrease, which will induce a loss of accuracy. Therefore, we recommend the use of SLDP according to the genetic architecture of the traits that are relevant in breeding practice. It is also important to improve the reliability of prior information, which could increase the sample size of the discovery population. Our GWAS results show that more significant sites and higher significance levels were obtained with an increase in sample size. In addition, the prior information obtained only from genome and phenotypic associations is insufficient. The results of the simulation GWAS proved that the QTN detection power of GWAS is limited, especially for low-heritability traits. Genomic functional annotation or clues provided by multi-omics data should be used to enrich the prior information.

However, some limitations of this study should be noted. First, our study was conducted in a single pig population only; hence, further investigations in other lines or species and multi-breed populations are necessary. Second, the identification of the optimal SLDP parameter required massive computation making it impossible to optimize the parameters for each generation in breeding practice. Therefore, the stability of these parameters necessitates further testing on a larger number of generations. If the parameters prove to be relatively stable across generations, one-time parameter optimization may be sufficient within a given generation. Finally, the performance of SLDP relies highly on the genetic architecture of the target traits, which could pose constraints on the application of this method in real breeding practice. We look forward to further research that explores and optimizes the applications of these techniques across diverse contexts.

## Conclusions

We developed selective linkage disequilibrium pruning, a marker selection method, to select trait-personalized marker sets from tens of millions of WGS data. This method increases the weight of candidate QTL regions in prediction models by taking prior information from GWAS into account. Our results showed that for real traits controlled by major QTL and for simulation traits controlled by a small number of QTN, SLDP performs better than the common LDP or SNP chips in the GBLUP and BayesR models; however, for traits not controlled by major QTL or those controlled by a massive number of minor QTL, there was no significant improvement in the prediction accuracy of SLDP. There are still some challenges to overcome in the application of SLDP, including the need for a more efficient and accurate identification of the predictive variants in the discovery set and the dependency on genetic architecture. In spite of these challenges, our results provide a valuable foundation for further research and applications of WGS-based genome selection.

### Supplementary Information

**Additional file 1: Figure S1.** Phenotypic distribution of the three real traits across the 3549 pigs included in this study.

**Additional file 2: Figure S2.** Principal component analysis (PCA) for the discovery, training and validation populations.

**Additional file 3: Figure S3.** Manhattan plot for AGE, BF and TTN in the discovery population, samples excluding the discovery population (the training and validation population) and all 3549 samples.

**Additional file 4: Table S1.** Number of retained SNPs after filtration based on different r^2^ thresholds.

**Additional file 5: Table S2.** Number of variants used in each SLDP scenario for AGE.

**Additional file 6: Table S3.** Number of variants used in each SLDP scenario for BF.

**Additional file 7: Table S4.** Number of variants used in each SLDP scenario for TTN.

**Additional file 8: Figure S4.** Histogram plot of the MAF frequency distribution for each data set.

**Additional file 9: Figure S5.** Heat map of genomic prediction accuracy for the simulated traits using SNPs after SLDP with r^2^ (from 1 to 0.05) and P-value (from 0.0001 to 0.01) gradients in the training population by BayesR.

**Additional file 10: Table S5.** Summary of the GWAS for six simulation scenarios based on different P-value thresholds in the discovery population.

## Data Availability

This published article and its supplementary information files include all data generated or analyzed during this study. Raw sequencing and phenotypic data for 2859 samples of the 3549 samples used in this study have been published in NCBI under accession nos. PRJNA681437 and PRJNA712489 and are available in the GigaScience repository, GigaDB (http://dx.doi.org/10.5524/100894). Phenotype and SNP data for additional samples are available from the corresponding authors upon reasonable request.
