# Supplementary material for: Genomic prediction based on selective linkage disequilibrium pruning of low-coverage whole-genome sequence variants in a pure Duroc population
Source: Genet Sel Evol. 2023 Oct 18;55:72. doi: 10.1186/s12711-023-00843-w (PMC10583454; doi:10.1186/s12711-023-00843-w)
Supplement: Supplementary file 1 — Additional file 1: Figure S1. Phenotypic distribution of the three real traits across the 3549 pigs included in this study. [file 12711_2023_843_MOESM1_ESM.docx]

**Additional File 1: Figure S1. Phenotypic distribution of the three real traits across the 3549 pigs included in this study.**


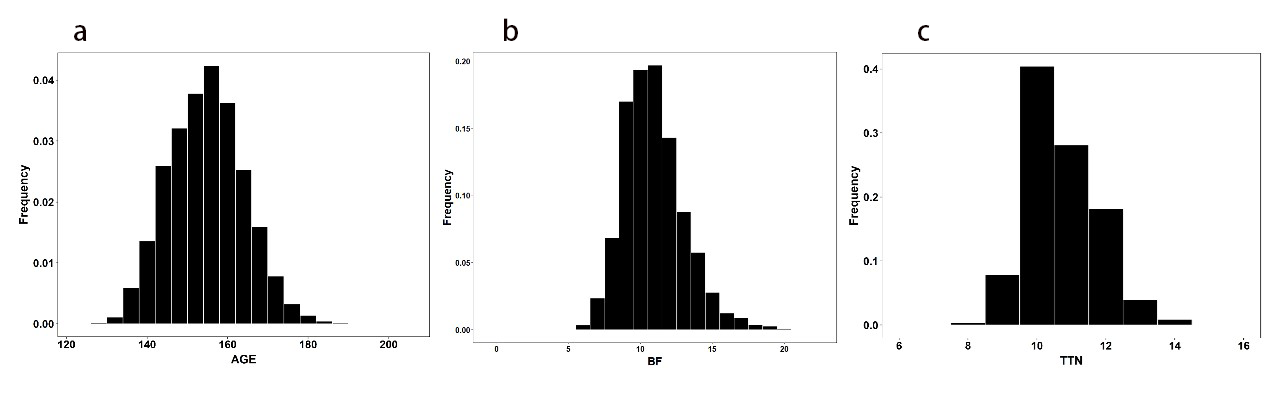


a, AGE; b, BF; c, TTN. AGE, age to 100 kg live weight; BF, back fat thickness; TTN, total teat number.
