# Supplementary material for: Genomic prediction based on selective linkage disequilibrium pruning of low-coverage whole-genome sequence variants in a pure Duroc population
Source: Genet Sel Evol. 2023 Oct 18;55:72. doi: 10.1186/s12711-023-00843-w (PMC10583454; doi:10.1186/s12711-023-00843-w)
Supplement: Supplementary file 2 — Additional file 2: Figure S2. Principal component analysis (PCA) for the discovery, training and validation populations. [file 12711_2023_843_MOESM2_ESM.docx]

**Additional File 2: Figure S2. Principal component analysis (PCA) for the discovery, training and validation populations.**

**
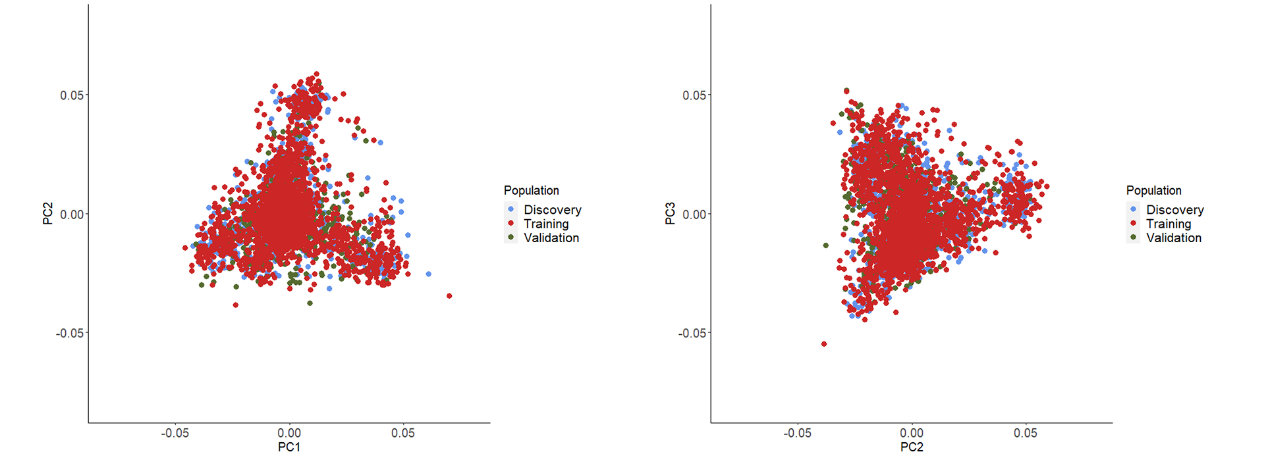
**

PC1, first principal component; PC2, second principal component; PC3, third principal component.
