# Supplementary material for: Genomic prediction based on selective linkage disequilibrium pruning of low-coverage whole-genome sequence variants in a pure Duroc population
Source: Genet Sel Evol. 2023 Oct 18;55:72. doi: 10.1186/s12711-023-00843-w (PMC10583454; doi:10.1186/s12711-023-00843-w)
Supplement: Supplementary file 3 — Additional file 3: Figure S3. Manhattan plot for AGE, BF and TTN in the discovery population, samples excluding the discovery population (the training and validation population) and all 3549 samples. [file 12711_2023_843_MOESM3_ESM.docx]

**Additional File 3: Figure S3. Manhattan plot for AGE, BF and TTN in the discovery population, samples excluding the discovery population (the training and validation population) and all 3549 samples.**

**
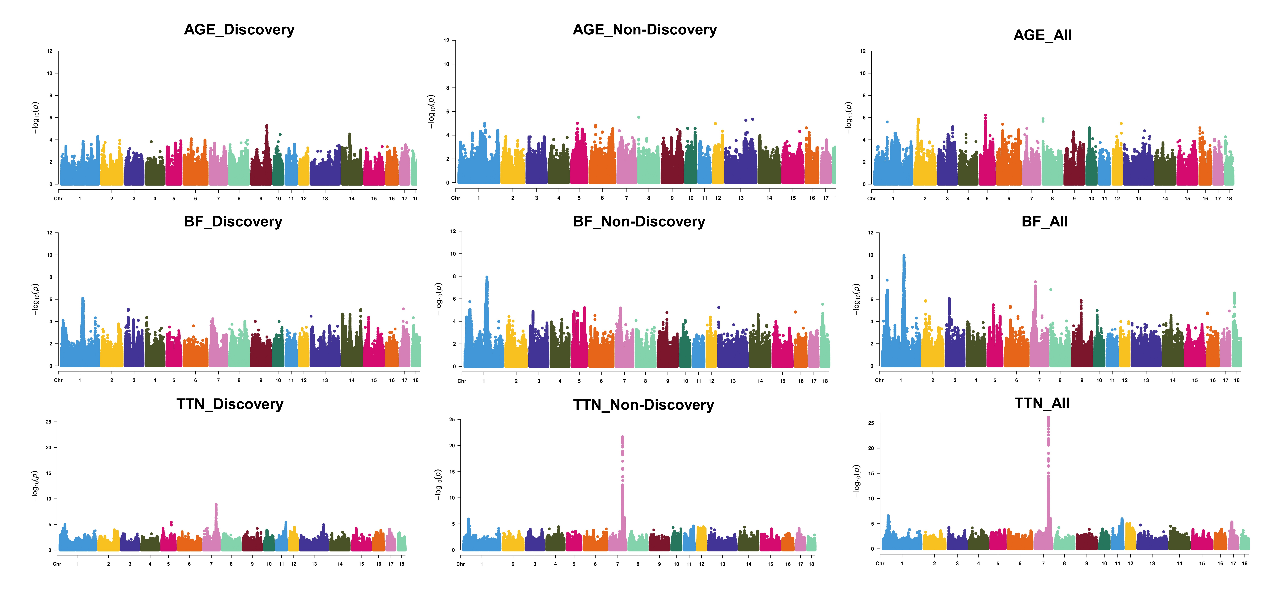
**

AGE, age to 100 kg live weight; BF, back fat thickness; TTN, total teat number.
