# Supplementary material for: Genomic prediction based on selective linkage disequilibrium pruning of low-coverage whole-genome sequence variants in a pure Duroc population
Source: Genet Sel Evol. 2023 Oct 18;55:72. doi: 10.1186/s12711-023-00843-w (PMC10583454; doi:10.1186/s12711-023-00843-w)
Supplement: Supplementary file 4 — Additional file 4: Table S1. Number of retained SNPs after filtration based on different r2 thresholds. [file 12711_2023_843_MOESM4_ESM.docx]

**Additional File 4: Table S1. Number of retained SNPs after filtration based on different r2 thresholds.**

| **LD threshold r^2^** | **Number of SNPs remained** |
| --- | --- |
| 1 | 1,823,847 |
| 0.99 | 361,865 |
| 0.95 | 158,265 |
| 0.9 | 124,679 |
| 0.85 | 104,912 |
| 0.8 | 89,891 |
| 0.75 | 77,567 |
| 0.7 | 67,490 |
| 0.65 | 58,532 |
| 0.6 | 51,016 |
| 0.55 | 44,118 |
| 0.5 | 38,057 |
| 0.45 | 32,580 |
| 0.4 | 27,756 |
| 0.35 | 23,554 |
| 0.3 | 20,007 |
| 0.25 | 16,831 |
| 0.2 | 14,142 |
| 0.15 | 11,739 |
| 0.1  0.05 | 9,736  7,984 |
