# Supplementary figures and images for: Genomic prediction based on selective linkage disequilibrium pruning of low-coverage whole-genome sequence variants in a pure Duroc population
Source: Genet Sel Evol. 2023 Oct 18;55:72. doi: 10.1186/s12711-023-00843-w (PMC10583454; doi:10.1186/s12711-023-00843-w)

**Additional File 8: Figure S4. Histogram plot of the MAF frequency distribution for each data set.**


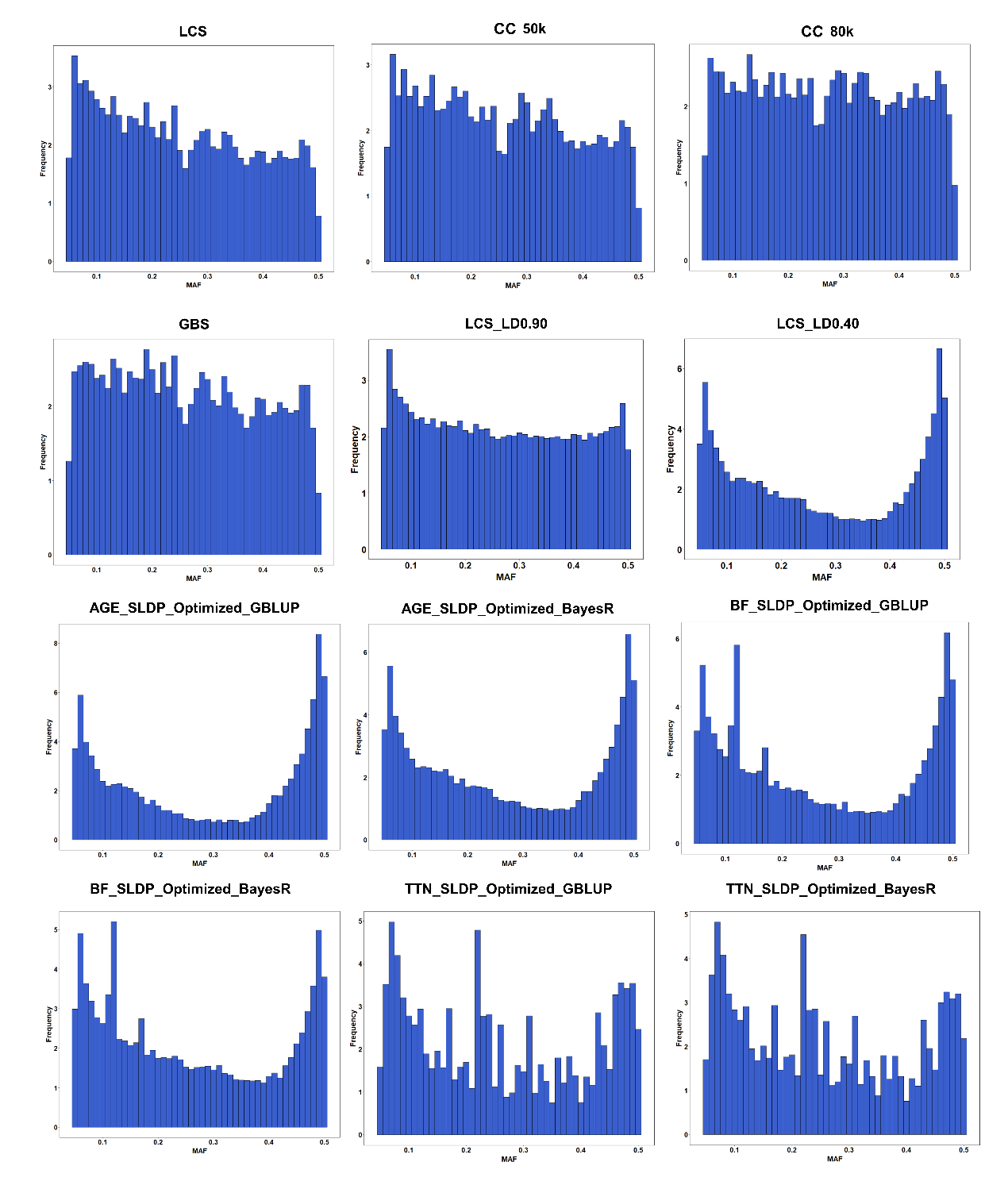

Supplement: Supplementary file 8 — Additional file 8: Figure S4. Histogram plot of the MAF frequency distribution for each data set. [file 12711_2023_843_MOESM8_ESM.docx]
