# Supplementary material for: Genomic prediction based on selective linkage disequilibrium pruning of low-coverage whole-genome sequence variants in a pure Duroc population
Source: Genet Sel Evol. 2023 Oct 18;55:72. doi: 10.1186/s12711-023-00843-w (PMC10583454; doi:10.1186/s12711-023-00843-w)
Supplement: Supplementary file 9 — Additional file 9: Figure S5. Heat map of genomic prediction accuracy for the simulated traits using SNPs after SLDP with r2 (from 1 to 0.05) and P-value (from 0.0001 to 0.01) gradients in the training population by BayesR. [file 12711_2023_843_MOESM9_ESM.docx]

**Additional File 9: Figure S5. Heat map of genomic prediction accuracy for the simulated traits using SNPs after SLDP with r2 (from 1 to 0.05) and P-value (from 0.0001 to 0.01) gradients in the training population by BayesR.**

**
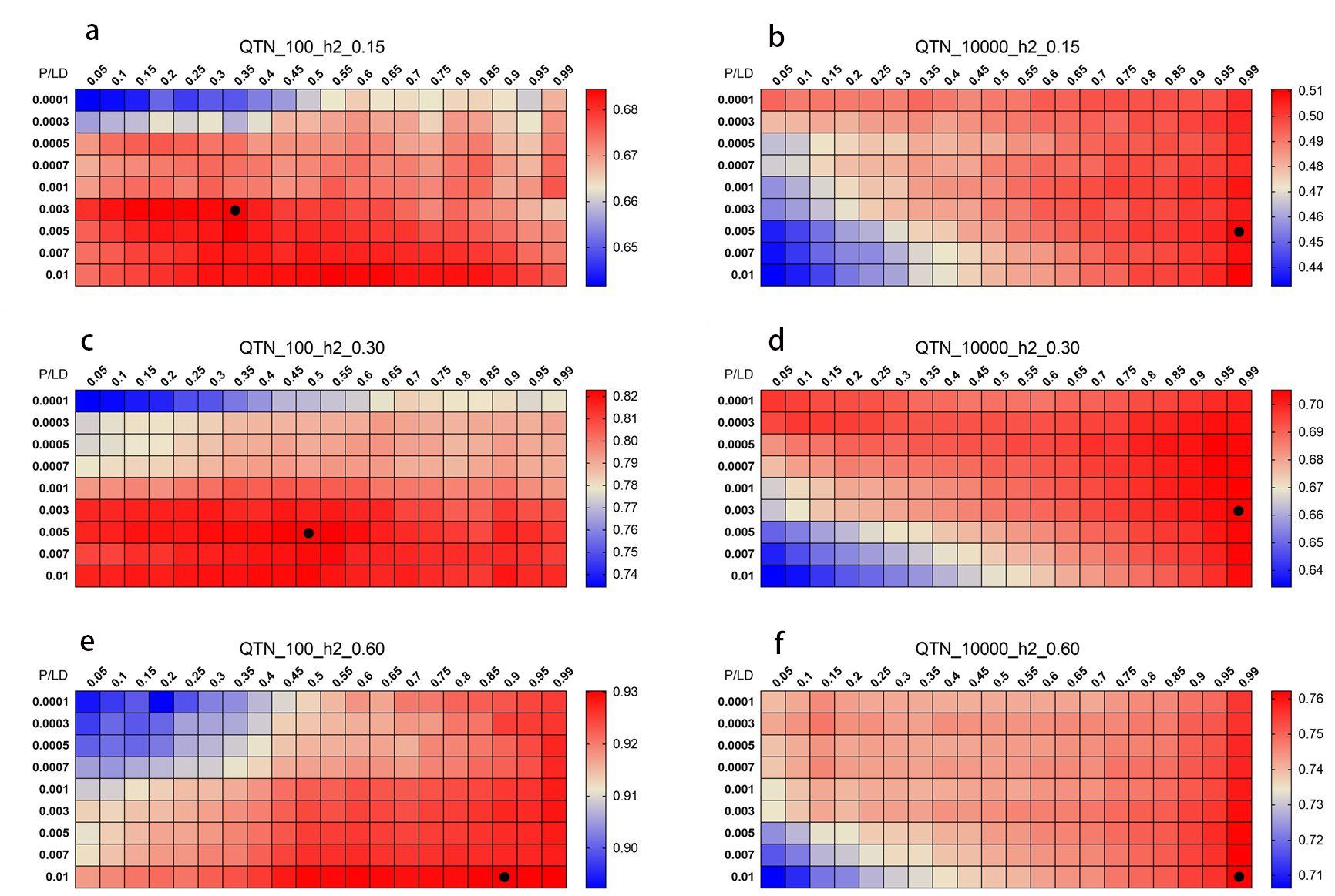
**

The results of BayesR model were showed in this figure; each square represents a parameter combination, while the accuracies are indicated with deeper colors corresponding to higher (red) or low (blue) accuracy. Black dots mark the optimal combination of parameters for accuracy. The accuracy was obtained by five-fold cross validation in training population and was defined as Pearson correlation between GEBV and TBV.
