# Supplementary material for: Genomic prediction based on selective linkage disequilibrium pruning of low-coverage whole-genome sequence variants in a pure Duroc population
Source: Genet Sel Evol. 2023 Oct 18;55:72. doi: 10.1186/s12711-023-00843-w (PMC10583454; doi:10.1186/s12711-023-00843-w)
Supplement: Supplementary file 10 — Additional file 10: Table S5. Summary of the GWAS for six simulation scenarios based on different P-value thresholds in the discovery population. [file 12711_2023_843_MOESM10_ESM.docx]

**Additional File 10: Table S5. Summary of the GWAS for six simulation scenarios based on different P-value thresholds in the discovery population.**

| **Scenarios** | **GWAS threshold P-value** | **Number of SNPs selected** | **Number of real QTN detected** | **Number of informative SNPs detected** | **Estimate false positive rate** |
| --- | --- | --- | --- | --- | --- |
| QTN_100_h2_0.15 | 0.0001 | 793 | 0 | 101 | 87.26% |
|  | 0.001 | 6,928 | 1 | 811 | 88.29% |
|  | 0.01 | 119,183 | 8 | 4454 | 96.26% |
|  | 0.05 | 531,436 | 22 | 10087 | 98.10% |
| QTN_100_h2_0.30 | 0.0001 | 1,370 | 2 | 241 | 82.41% |
|  | 0.001 | 14321 | 5 | 2330 | 83.73% |
|  | 0.01 | 111,937 | 16 | 6153 | 94.50% |
|  | 0.05 | 515,817 | 32 | 16557 | 96.79% |
| QTN_100_h2_0.60 | 0.0001 | 28,072 | 7 | 5312 | 81.08% |
|  | 0.001 | 47,382 | 12 | 8264 | 82.56% |
|  | 0.01 | 155,994 | 22 | 16768 | 89.25% |
|  | 0.05 | 478,020 | 31 | 22673 | 95.26% |
| QTN_10000_h2_0.15 | 0.0001 | 642 | 0 | 111 | 82.71% |
|  | 0.001 | 11,226 | 13 | 4987 | 55.57% |
|  | 0.01 | 97,195 | 107 | 33410 | 65.63% |
|  | 0.05 | 478,419 | 559 | 168330 | 64.82% |
| QTN_10000_h2_0.30 | 0.0001 | 1,206 | 4 | 695 | 42.37% |
|  | 0.001 | 10,312 | 17 | 3968 | 61.52% |
|  | 0.01 | 108,874 | 129 | 38804 | 64.35% |
|  | 0.05 | 514,266 | 569 | 181029 | 64.80% |
| QTN_10000_h2_0.60 | 0.0001 | 2,563 | 6 | 996 | 61.14% |
|  | 0.001 | 12,160 | 23 | 4891 | 59.78% |
|  | 0.01 | 113,145 | 165 | 52393 | 53.69% |
|  | 0.05 | 499,725 | 620 | 196972 | 60.58% |
